# Factors associated with the oral health-related quality of life of patients with temporomandibular disorder at the final follow-up visit: a cross-sectional study

**DOI:** 10.1038/s41405-022-00122-8

**Published:** 2022-10-02

**Authors:** Prangtip Potewiratnanond, Nareudee Limpuangthip, Varangkana Karunanon, Ansaya Buritep, Athitaya Thawai

**Affiliations:** 1 Department of Occlusion, Faculty of Dentistry, Chulalongkorn University, Bangkok, Thailand; 2 Department of Prosthodontics, Faculty of Dentistry, Chulalongkorn University, Bangkok, Thailand; 3 Faculty of Dentistry, Chulalongkorn University, Bangkok, Thailand

**Keywords:** Occlusion, Prosthetic dentistry

## Abstract

**Objectives:**

To determine the oral health-related quality of life (OHRQoL) of temporomandibular disorder (TMD) patients at the final follow-up visit, and to investigate the associated factors.

**Materials and methods:**

This cross-sectional study comprised 227 TMD patients. Dependent variable was OHRQoL determined by telephone interview using the 14-item Oral Health Impact Profile (OHIP-14) questionnaire after the final follow-up visit. Independent variables were collected from dental records, comprising age, sex, treatment duration, diagnosis, clinical parameters (mouth-opening distances), and pain perception. TMD patients were diagnosed as having masticatory muscle disorders (TMDM), temporomandibular joint (TMJ) disorders (TMDJ), or combined muscle and TMJ disorders (TMDC). Bivariate analyses and multivariable linear regression were used to analyze the factors associated with OHIP-14 scores.

**Results:**

Bivariate analyses demonstrated higher OHIP-14 scores in younger patients, females, having TMDC, and lower mouth-opening distance. Multivariable analysis demonstrated the association of higher OHIP-14 scores with being younger and having TMDC. Participants with TMDC demonstrated greater improvement in unassisted mouth-opening distance, compared with the other clinical diagnosis groups.

**Conclusions:**

At the final follow-up visit, oral health impact problems were reported mainly in physical pain and psychological discomfort domains. Better OHRQoL was found in older, and TMDM or TMDJ patients.

## Introduction

Temporomandibular disorders (TMDs) are a group of musculoskeletal and neuromuscular conditions involving the temporomandibular joints (TMJ), masticatory muscles, and all associated tissues [1]. An epidemiological study revealed that the prevalence of TMD signs and symptoms varies widely due to the disparities among populations, and the use of different methods and clinical criteria between studies [2]. A longitudinal study in an adult population reported that the trend of TMD symptoms increased over the 20-year period from 27% to 38% [3].

According to the Diagnostic Criteria for TMD (DC/TMD), TMDs can be classified into muscle and joint origin that are divided into 12 common diagnoses [4]: (1) masticatory muscle disorders (TMDM), which consist of diagnoses of myalgia, local myalgia, myofascial pain, myofascial pain with referral and headache attributed to TMD; (2) TMJ disorders (TMDJ), i.e., diagnoses of arthralgia, disc displacement with reduction, disc displacement with reduction with intermittent locking, disc displacement without reduction with a limited opening, disc displacement without reduction without limited opening, and degenerative joint disease and subluxation.

The current TMD etiology is a multifactorial biopsychosocial concept. Therefore, TMD management comprises physical and psychological approaches. Conventional therapies are preferred, including self-care instruction, occlusal splint, occlusal adjustment, and mandibular manipulation [5–10]. The optimal evaluation of TMD treatment outcome should be based on clinical parameters and patient-reported outcomes [11]. The clinical parameters consist of the clinician’s evaluation of a patient’s function, such as the range of mandibular movement, jaw function, and pain on muscle palpation [4, 12]. The patient-reported outcomes commonly include pain intensity level, psychological impairment, and oral health-related quality of life (OHRQoL) [6, 13–16]. The OHRQoL determines the impact of an oral condition on a person’s ability to perform physical, psychological, and social functions that reflect oral health and well-being [17].

Several studies have reported the OHRQoL of TMD patients and its related factors [14, 18–21]. Patients with TMD commonly have lower OHRQoL, compared with healthy individuals [21, 22]. The OHRQoL of TMD patients is influenced by demographic variables, pain intensity, jaw function, and psychological impairment [14, 18–20]. However, these studies evaluated the OHRQoL and its associated factors prior to TMD treatment. Few studies have investigated the impact of a person’s characteristics and clinical improvement on TMD patients’ OHRQoL after treatment [13, 23, 24]. Therefore, the objective of this study was to determine the OHRQoL of TMD patients at the final follow-up visit, and investigate the associated factors.

## Materials and methods

The present cross-sectional study was conducted from October 2021 to March 2022. The patients were contacted via telephone from October to December 2021. The participants were 227 TMD patients who received TMD treatment, and had regular follow-up visits at the Department of Occlusion, Chulalongkorn University Dental Hospital from 2016 to 2020. The TMD treatment consisted of self-care instruction and occlusal splint therapy. In approximately 5% of cases, occlusal adjustment and mandibular manipulation were additionally provided when the conservative approaches could not alleviate the patient’s symptoms. The inclusion criteria were TMD patients who had received TMD treatment, could communicate in Thai, and had at least two follow-up visits after occlusal splint delivery. A previous systematic review and meta-analysis showed that the TMD symptoms were generally improved after three months of occlusal splint therapy [5], which encompasses the first date of TMD treatment through the two follow-up visits in our dental school. The exclusion criteria were patients who were unable to respond to the questionnaire, or could not be contacted via telephone. The study protocol was approved by the Human Research Ethics Committee of the Faculty of Dentistry, Chulalongkorn University (HREC:2021-062). The subjects provided informed consent permission via telephone interview prior to participating in the study.

### Dependent variable

The dependent variable was the OHRQoL assessed after the final follow-up visit. The participants were telephone interviewed about their OHRQoL using the validated Thai version of the Oral Health Impact Profile (OHIP-14) index [25]. The questionnaire consisted of 14 items within 7 domains, functional limitation, physical pain, psychological discomfort, physical disability, psychological disability, social disability, and handicap. The participants gave responses on the impact frequency using a 5-point ordinal scale (0—never, 1—hardly ever, 2—occasionally, 3—fairly often, and 4—very often). The summed OHIP-14 scores ranged from 0 to 56; higher scores reflected a poorer OHRQoL.

### Independent variables

The participants’ information was collected from their dental hospital records. The baseline data comprised age, sex, first treatment date and final follow-up date, and clinical diagnosis. For the clinical diagnosis, the participants were categorized into three groups according to the DC/TMD axis I protocol: (1) TMDM; (2) isolated TMDJ; and (3) a combined muscle and joint disorder (TMDC). The treatment duration from the first treatment date to the final follow-up visit was categorized into less than 6 months, 6 months to less than 1 year, and 1–5 years.

At the baseline and the final follow-up visits, two clinical parameters were recorded: maximum unassisted and maximum assisted mouth-opening distances (mm). At the final follow-up visit, the participants were interviewed face-to-face about their pain perception. The participants rated their pain intensity level using a 10-point ordinal scale; a higher score indicated greater pain. In addition, the participants gave responses on their subjective change in pain, whether it was better, similar, or worse than that of pre-treatment.

Power analysis of the sample size was calculated using G*Power 3.1.9.4 software. Based on the hypothesis that the OHIP-14 scores in the three TMD diagnosis groups were significantly different, the F-test and ANOVA fixed effect, omnibus, one-way was used. We found that the mean OHIP-14 scores of the TMDM (*n* = 70), TMDJ (*n* = 76), and TMDC (*n* = 81) groups were 4.2, 4.2, and 7.3, respectively, and the standard deviation within each group was 6.0, the effect size value of 0.25 was calculated. Using a 5% type-I error and two-tailed test, a 92.2% power was achieved.

### Statistical analysis

The data were analyzed using STATA software version 14.0 at the 5% significance level. Descriptive statistics were performed to calculate the mean and 95% confidence interval, as well as the frequency and percentage distribution. The associations between the OHIP-14 scores and each independent variable were determined using bivariate analyses. The independent *t*-test and one-way analysis of variance were used to evaluate the differences in the OHIP-14 scores between each categorical variable, whereas the association between the OHIP-14 score and a continuous variable was evaluated using Pearson’s correlation. The variables with a *p-*value < 0.20 were included in the multivariable analysis. Multivariable linear regression was used to analyze the factors associated with the OHIP-14 scores. The variance inflation factor (VIF) of each factor were identified to determine the multicollinearity of the variables, and the factors with a VIF >5 were excluded from the regression model. The associations between the clinical diagnosis and mouth-opening distances, adjusting for age and sex, were further determined using a multivariable linear regression.

## Results

**Fig. 1:**
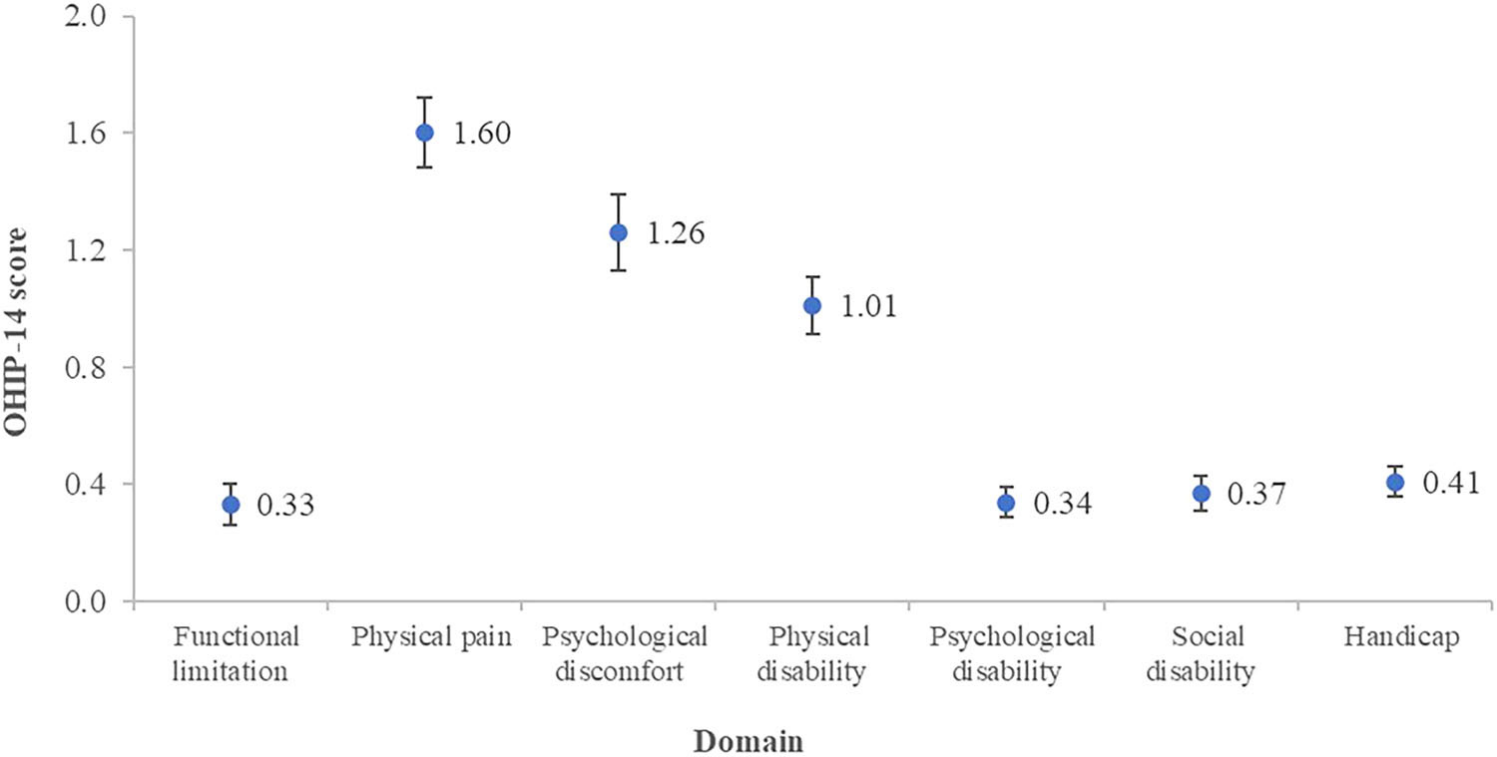
Dot-and-whisker plot of OHIP-14 score represented in seven domains. The dot and whisker indicated the mean and standard error, respectively.

The mean age of the TMD participants was 36.34 years old (range 13–75 years old) with a male:female ratio of 1:3.28 (Table 1). The mean OHIP-14 score was 5.31, ranging from 0 to 27. The highest OHIP-14 scores were on physical pain and psychological discomfort domains (Fig. 1). The bivariate analyses revealed that the OHIP-14 score was significantly higher in the TMD patients of younger age, female, being diagnosed as having TMDC, and had a lower mouth-opening distance at baseline (Table 1).

**Table 1:** Participant characteristics and bivariate analysis of the association between OHIP-14 score and independent variables.

Variables	Overall (*N* = 227)	OHIP score: mean (95%CI)	*p*-value
Age (years), mean (95% CI)	36.3 (34.3, 38.4)	*r* = −0.15*	0.02
Sex, *n* (%)			
Male	53 (23.3)	3.6 (2.3, 4.9)	0.01
Female	174 (76.7)	5.8 (4.9, 6.7)	
*Baseline variables*			
Clinical diagnosis, *n* (%)			
Muscle (TMDM)	70 (30.8)	4.2 (2.9, 5.5)	
TMJ (TMDJ)	77 (33.9)	4.2 (3.0, 5.4)	0.01
Combined (TMDC)	80 (36.3)	7.3 (5.8, 8.9)	
Clinical parameters, mean (95% CI)			
Maximum unassisted mouth-opening distance (mm)	46.3 (45.3, 47.2)	*r* = −0.14*	0.04
Maximum assisted mouth-opening distance (mm)	48.6 (47.7, 49.6)	*r* = −0.15*	0.03
*After treatment variables*			
Subjective change in pain compared with baseline, *n* (%)			
Better	155 (68.2)	6.4 (3.4, 13.7)	
Same	62 (27.3)	4.3 (2.8, 5.7)	0.13
Worse	10 (4.5)	5.7 (4.5, 6.8)	
Clinical parameters, mean (95% CI)			
Maximum unassisted mouth opening (mm)	43.9 (42.9, 44.9)	*r* = 0.09	0.24
Maximum assisted mouth opening (mm)	47.4 (46.5, 48.3)	*r* = 0.10	0.20
Changes in maximum unassisted mouth opening (mm)	2.9 (2.2, 3.6)	*r* = 0.08	0.27
Changes in maximum assisted mouth opening (mm)	1.7 (1.0, 2.3)	*r* = 0.10	0.20
Pain intensity score (0–10)	1.3 (1.0, 1.6)	*r* = 0.10	0.16
*Treatment durations, n (%)*			
<6 months	85 (39.2%)	5.23 (3.97, 6.51)	
6 months to 1 year	69 (30.4%)	4.25 (3.02, 5.47)	0.09
>1–5 years	69 (30.4%)	6.48 (4.80, 8.15)	

*CI* confidence interval, *r* Pearson correlation coefficient.

*p < 0.05.

The variables with *p* < 0.20, subjective change in pain, pain intensity level, and treatment duration, were additionally included in the multivariable analysis. Because the maximum assisted mouth-opening distance had a VIF >5, the variable was removed from the final regression model. The multivariable regression analyses demonstrated a significant association between a higher OHIP-14 score and younger age and clinical diagnosis of TMDC (Table 2). Moreover, the participants who were diagnosed as having TMDC demonstrated greater improvement in unassisted mouth-opening distance, compared with the other clinical diagnosis groups (Table 3).

**Table 2:** Multivariable linear regression analysis of the association between OHIP-14 score and independent variables.

Variables	OHIP-14 score: adjusted beta (95% CI)	VIF
Age (years)	−0.06 (−0.11, −0.002)*	1.14
Sex		
Male	0 (ref)	1.18
Female	1.59 (−0.70, 3.85)	
*Baseline variables*		
Clinical diagnosis		
Muscle	0 (ref)	
TMJ	−0.92 (−3.20, 1.36)	1.36
Mixed	3.11 (0.95, 5.27)*	1.40
Clinical parameters		
Maximum unassisted mouth opening (mm)	−0.02 (−0.21, 0.16)	2.51
*After treatment variables*		
Subjective change in pain compared with baseline		
Same	0 (ref)	
Better	1.04 (−1.06, 3.14)	1.25
Worse	1.36 (−3.38, 6.10)	1.26
Pain intensity score (0–10)	0.31 (−0.15, 0.78)	1.21
*Treatment durations*		
<6 months	0 (ref)	
6 months to 1 year	−1.26 (−3.51, 0.99)	1.29
>1–5 years	1.05 (−1.19, 3.29)	1.37

*beta* beta-coefficient, *VIF* variance inflation factor.

*p < 0.05.

**Table 3:** Multivariable linear regression analysis of the association between changes in clinical parameters and clinical diagnosis.

Clinical diagnosis	Adjusted beta-coefficient (95% CI)	
	Changes in unassisted mouth opening (mm)	Changes in assisted mouth opening (mm)
TMDM	0 (ref)	0 (ref)
TMDJ	0.37 (−1.36, 2.10)	0.26 (−1.37, 1.88)
TMDC	1.78 (0.04, 3.53)*	0.58 (−1.05, 2.22)

Adjusting for age and sex.

*p < 0.05.

## Discussion

Our results indicated that OHRQoL problems were reported by TMD patients at the follow-up visit but with a low-intensity scale. A lower OHRQoL was more frequently reported by younger TMD patients, and those diagnosed as having TMDC, compared with those having TMDM or TMDJ.

Several OHRQoL indices have been used to evaluate TMD patients, such as the Geriatric Oral Health Assessment Index [26], Child Perceptions Questionnaires [27], and OHIP [21]. A systematic review reported that the OHIP-14 is one of the most frequently used OHRQoL indices to determine the efficacy of TMD treatment because it has optimal psychometric properties, and has been translated into several languages for use worldwide [28].

Although oral health impacts were present after TMD treatment, the problems were typically infrequent with an average score of 1–2 for each domain. Furthermore, according to a previous study in our dental school [22], the mean baseline OHIP-14 score of TMD patients before treatment (mean ± SD) was 24.4 ± 12.2. Consistent with a previous study, the OHIP-14 score of the TMD patients was higher than the healthy control [21]. Our finding showed that the OHIP-14 score post-treatment was only 5.31, which was similar to that of patients without TMD who went for routine dental check-ups [22]. Based on these findings, we hypothesize that most of the patients in the present study might have better OHRQoL after TMD treatment. In our dental school, an occlusal splint and self-care instruction are provided for TMD patients as a conservative approach. As supported by several studies [5, 13, 23, 29], an occlusal splint with self-care instruction is an effective approach to improve the OHRQoL of patients with mild-to-moderate TMD symptoms.

Our finding demonstrated that the major oral health impacts after TMD treatment were reported for physical pain and psychological discomfort domains. These domains consist of pain, eating difficulty, feeling stress, and personal appearance concern. This was consistent with previous studies that evaluated the OHRQoL of patients prior to receiving any TMD treatment [21, 22, 30]. Our findings indicate that similar OHRQoL problems were found after conventional TMD treatment, but with lower intensity.

In the present study, better OHRQoL was reported in older patients. Females tended to have a poorer OHRQoL, however, the result was not significant in the multivariable analysis. Our findings were consistent with epidemiologic studies that revealed that TMD signs and symptoms were more frequently reported in females, middle-aged, and those with a poorer psychological status [2, 28] In addition, poorer health-related quality of life was more frequently reported in younger age [16], and female TMD patients [30, 31]. The difference between males and females might be due to different biological, physiological, and behavioral factors.

A poorer OHRQoL was more frequently reported in TMD patients with TMDCs. This finding is consistent with a previous study by Almoznino et al. that reported that the patients with combined muscle and TMJ symptoms had the poorest OHRQoL prior to TMD treatment, compared with those having muscle or joint disorder alone [21]. This might be because the combined muscle and TMJ symptoms affect more anatomical structures, resulting in more pain and discomfort. Although a poorer OHRQoL was reported in TMDC patients, they demonstrated the greatest improvement in their unassisted mouth-opening distance. Therefore, TMD evaluation should consider both patient-reported and clinical parameters. Further study is suggested to develop additional or alternative TMD treatments to improve the OHRQoL of the patients with combined TMD symptoms.

The OHRQoL of TMD patients who underwent different treatment durations was similar. In this study, the OHRQoL was investigated in TMD patients who had received treatment for at least 3 months and enrolled in the maintenance phase. Therefore, a relatively stable OHRQoL was assumed. Our results are supported by previous studies that found that conservative treatment reduced the TMD symptoms within 30 days to 6 months after treatment begins [13, 23, 24], and self-perceived OHRQoL did not change after 6 months [13]. This might be the reason why the OHRQoL of the TMD patients was not associated with treatment duration.

The present study demonstrated the OHRQoL outcome at the final follow-up visit after TMD treatment with up to a 5-year follow-up in the dental school, which is the main center for TMD treatment in Thailand. However, some limitations exist. Due to its cross-sectional study design, a cause-effect relationship between the underlying determinants and OHRQoL cannot be concluded because the patients’ OHIP-14 score before TMD treatment was unavailable. Some confounding factors that might affect the outcome of TMD treatment, such as obesity and stress level [14, 32], were not investigated. Further studies should be conducted to determine the effectiveness of the treatment approaches for a causal-effect relationship and to identify the appropriate treatment approach for different TMD diagnoses in the OHRQoL aspect.

## Conclusion

Our findings indicated that oral health impact problems were reported at the follow-up visit, mainly in physical pain and psychological discomfort domains. A better OHRQoL after TMD treatment was associated with older patients and those who were diagnosed as having only muscle or TMJ symptoms.
